# Recognition and management of agitation in acute mental health services: a qualitative evaluation of staff perceptions

**DOI:** 10.1186/s12912-020-00495-x

**Published:** 2020-11-10

**Authors:** Joshua Tucker, Lisa Whitehead, Peter Palamara, Josephine Xenia Rosman, Karla Seaman

**Affiliations:** 1 Albany Health Campus, 30 Warden Avenue, Spencer Park, Western Australia 6330 Australia; 2 Centre for Nursing, Midwifery and Health Services Research, School of Nursing and Midwifery, Edith Cowan University, Building 21, Level 4, 270 Joondalup Drive, Joondalup, Western Australia 6027 Australia

**Keywords:** Agitation, Assessment, Management, Mental health, Nurses, person-centred care, Qualitative research

## Abstract

**Background:**

Agitation among patients is a common and distressing behaviour across a variety of health care settings, particularly inpatient mental health. Unless recognised early and effectively managed it can lead to aggression and personal injury. The aim of this paper is to explore the experiences of mental health nurses in recognising and managing agitation in an inpatient mental health setting and the alignment of these experiences with best practice and person-centred care.

**Methods:**

This study used a descriptive qualitative methodology. Semi-structured focus group interviews were conducted with 20 nurses working in a mental health unit in 2018. Nursing staff described their experiences of assessing and managing agitation. Descriptive and Thematic Analysis were undertaken of the transcribed focus group dialogue.

**Results:**

Nurses combined their clinical knowledge, assessment protocols and training with information from patients to make an individualised assessment of agitation. Nurses also adopted an individualised approach to management by engaging patients in decisions about their care. In keeping with best practice recommendations, de-escalation strategies were the first choice option for management, though nurses also described using both coercive restraint and medication under certain circumstances. From the perspective of patient-centred care, the care provided aligned with elements of person-centred care nursing care.

**Conclusion:**

The findings suggest that clinical mental health nurses assess and manage agitation, with certain exceptions, in line with best practice and a person-centred care nursing framework.

## Background

Agitation among patients is a frequently cited behavioural problem across a variety of health settings [1]. While it is considered to be distinctly different to aggression [2], without timely assessment and management it can quickly escalate to a loss of personal control, aggression and violence [1] and result in injury to patients and staff [3, 4]. Agitation can also lead to increased periods of hospitalisation [5] and episodes of readmission [6] resulting in increased health care costs [5, 7].

There is limited epidemiologic evidence on the prevalence of agitation in mental health settings. Estimates range between a high of 47.5% among newly hospitalised adults with schizophrenia in China [8] to a low of 4.6% among psychiatric emergency presentations in Europe [9]. This variation is due in part to the ongoing debate over a standardised definition of agitation and the use of appropriate measurement scales to aid the assessment of agitation in the clinical setting [10].

The causes of and triggers for an episode of agitation are complex. They include psychiatric conditions such as schizophrenia and dementia; substance abuse (including legitimate use of medications); medical or physiologic disorders such as traumatic brain injury and drug toxicity [11]. Other risk factors for an episode of agitation among patients in a mental health setting include a pre-admission history of aggression and involuntary admission to hospital [4, 8] and presenting as aggressive or impulsive at admission [8, 12].

Managing episodes of agitation requires prompt, safe intervention to de-escalate the behaviour to minimise the risk of physical injury and the need to use more invasive, coercive measures should the patient become aggressive [4, 13]. The best practice recommendation for mild-moderate agitation is for the ‘first choice’ use of non-pharmacological interventions such as verbal de-escalation or engaging the patient in diversionary activities [1, 13]. Best practice also recommends the avoidance of invasive measures such as physical or mechanical restraint and environmental seclusion of the patient due to their potential to result in physical injuries [13] and psychological distress [14, 15] to staff and patients and to negatively impact the therapeutic relationship between the two [4]. In circumstances where de-escalation or diversionary strategies are ineffective or inappropriate, the best practice pharmacological treatment of agitation is to use medications that are safe, easy and quick to administer; have rapid impact, and do not overly sedate the patient [4].

One of the many challenges for nursing staff in managing acute agitation is to deliver optimal and effective care while preserving the patient’s dignity and right to participate in the decision making of their care, each of which are central to the practice of person-centred care (PCC) in the mental health setting [16]. PCC is a highly valued and widely practiced approach in mental health care as it aims to address the many short comings of a strict biomedical approach by considering the person and their illness holistically [17, 18]. In particular, PCC focuses on the development of a quality, respectful and collaborative relationship between the health care provider and the patient [17]. This means that nurses should explore and consider patient beliefs and values about their health and treatment and take these into account in shared decision making over treatment choices that focus on the person in a broader sense and not just their medical requirements [17]. If these processes are threatened, for example if the agitated patient is restrained, secluded or medicated without their agreement to manage their condition, the outcomes of PCC such as involvement with care planning and satisfaction with care [17] may be compromised. Person-centred mental health care must therefore balance managing risk and self-determination and the tension between delivering evidenced-based, cost effective care and patient choice [19].

The aims of this qualitative study are to develop an understanding of mental health nurses’ experiences in recognising and managing agitation among inpatients, and secondly, to consider the findings in relation to best practice principles for managing agitation and PCC principles and processes.

## Methods

### Design, sample and setting

A qualitative, cross-sectional, descriptive approach was employed to address the study aims. Eligible participants were the clinical nursing staff of an adult mental health unit rostered for work on the days of data collection. All rostered staff were purposely recruited to participate in the focus group discussions and all agreed to participate. A total of 20 clinical nurses (15 females, 5 males) participated in four focus groups each with five participants. Their clinical experience in a mental health setting ranged between 1 year and 40 years (median 7 years). Around half of all participants had prior experience working in a general nursing setting.

### Data collection

Consenting participants were non-randomly assigned to one of four semi-structured focus group interviews conducted during the day on 1 day in April, 2018. Each focus group was facilitated by one of the authors (JT) and a member of the data collection research team named in the Acknowledgements (JR, AD, MA, AA, MN). All focus group facilitators were Master of Nursing post-graduate entry students. A research mentor (KS) also attended all focus group interviews, along with another member of the data collection research team who managed the audio recording and took notes on the group interactions. The focus group interviews were conducted in a room within the hospital’s Mental Health Unit during working hours*.* At the start of each session participants were reminded of the need to respect the privacy and confidentiality of all group participants and that the group discussion would be audio recorded and augmented with the observer’s notes on the participants’ responses. The facilitator also reiterated the study’s aims and objectives. Six questions (Table 1) were used to frame the group discussion of around 30 min duration.

**Table 1:** Nursing staff focus group questions on patient agitation

1. What signs and symptoms do you recognise as indicating agitation in a patient?	
2. Can you briefly describe an incident of agitation you have been involved with in the ward and your response to this?	
3. What non-pharmacological and pharmacological interventions do you use? Can you talk us through an example? Is the example have given typical of how you would manage an episode of agitation?	
4. Have you been offered or encouraged to take up training to help deal with agitation? If yes, could you describe the training you received? If not, what sort of training do you think would be beneficial to dealing with agitation?	
5. What are your thoughts on the current policy and procedures for assessing and managing agitation?	
6. Do you have any further feedback or comments in regards to recognising and managing agitation in the patients in the ward setting?	

### Ethical considerations

Staff were provided with a Participant Information Study sheet which outlined the aims and requirements of the study and the ethics approvals obtained from the university and the hospital for the study. Study participants signed an Informed Consent form to acknowledge the requirements and terms of participation, including the audio recording of the focus group discussion.

### Data analysis

Thematic Analysis (TA) was used to analyse the prepared transcripts. TA was selected because the methodology facilitates the identification of repetitive ideas, topics and themes for the purpose of classifying responses (data) into thematic categories [20, 21]. All data, without exclusions, were coded by members of the data collection research team and two of the four named authors (JT, LW) individually. Themes were subsequently identified from the assigned codes and categorisations. Following this, members of the research team held several discussions to comparatively analyse the data and emerging themes from which more themes and sub-themes were developed. Any disagreements were resolved as a group. The themes and sub-themes emerged and evolved until the team was satisfied that all relevant themes had been exhaustively identified.

### Rigour

Trustworthiness and quality was established through the steps of credibility, transferability, dependability, confirmability, and authenticity proposed by Lincoln and Guba [22]. Data segments were sorted, categorized, summarized, and then organized into labels and themes. All authors reviewed verbatim transcripts and discussed the coding and themes until consensus was reached. Credibility of the data was established through sampling of the whole clinical team, the use of field notes and achieving saturation. Transferability was achieved by exploring the interview data in the context of confirming evidence across the focus groups. Communication between team members was open during the review of transcripts and thematic interpretation in working toward dependability. Confirmability was sought by linking interpretations with participants’ quotes.

## Results

**Fig. 1:**
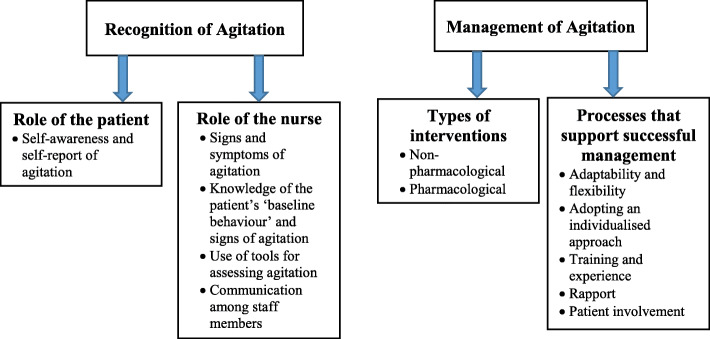
Identified main themes and subthemes from the focus group interviews

Two major themes were generated: the recognition of agitation and the management of agitation. A number of subthemes were generated within each major theme (Fig. 1).

### Recognition of agitation

Two themes were identified from the nurses’ accounts of the recognition of agitation: the role of the patient and the role of the nurse. The nurses also described these roles as having a ‘symbiotic’ relationship.

#### The role of the patient

##### Self-awareness and self-report of agitation

Nurses described the patients’ own identification of their level of agitation as playing a key role in the recognition of agitation. One nurse described that is was common for patients to approach nurses to let them know they were becoming agitated, pre-empting the nurse’s assessment.

*A lot of the time the patients will come up and say, I need some medication, I’m agitated.* (Group C)

Another nurse commented that it was in the patients’ best interests that they self-reported increased feelings of agitation to assist with early intervention.

*If someone identifies that they are getting aroused or getting agitated, it is probably the best thing for them to approach the nurses.* (Group A)

Nurses also described the completion of the ‘Coping and Safety Plan’ on admission by the patient as a valuable form to assist nurses in gathering information about symptoms, triggers, and management strategies.

#### The role of the nurse

Within this theme, four sub-themes were identified: clinical knowledge of the signs and symptoms of agitation; knowledge of the patient’s ‘baseline behaviour’ and signs of agitation; the use of tools for assessing agitation, and communication among staff members.

##### Clinical knowledge of the signs and symptoms of agitation

Nurses described various signs and symptoms related to agitation. In every focus group pacing, restlessness and raised voices were identified. Banging on the nurses’ station or slamming doors was mentioned in two groups, while another group said agitated patients could be uncooperative and engage in anti-social behaviour. Symptoms such as pallor, clenched fists, exaggerated hand gestures, sweating, and tightly pursed mouths were also described as signs of agitation in patients. The nurses described their observation of patients’ behaviour and physical activity and their ability to act quickly to diffuse agitation when signs and symptoms arose.

*It’s about being able to observe closely and then pick up on the early warning signs earlier.* (Group D)

##### Knowledge of the patient’s ‘baseline behaviour’ and signs of agitation

Nurses acknowledged that patients are diverse and as such display different signs and symptoms of agitation and reasons for becoming agitated. Knowing and understanding each patient was described as aiding the recognition of the onset of agitation.

*Those kind of things are obvious ones but there are much more subtle ones, if you know someone’s baseline and the way they behave when they are not agitated you start to see signs of them changing in their behavior.* (Group D)

Nurses reported that they used the patient history and the ‘Coping and Safety Plan’ to gain an understanding of the baseline behaviour of the patient and establish an “early warning sign” of agitation and how to manage agitation. Even so, one nurse acknowledged that agitation can occur without such warning signs.

*So, identifying early warning signs of people's agitation is very important and what we focus on in the training.* (Group D*)*

##### The use of tools for assessing agitation

Nurses described the different assessment tools used in the mental health unit to assess risk and measure the degree of agitation in patients. The ‘Brief Risk Assessment Tool’ was used on the wards to identify whether patients were at high or low risk of agitation based on their history. Nurses also acknowledged the use of a ‘Targeted Risk Assessment’ tool, which is a new initiative within the psychiatric intensive care unit (i.e., the locked ward) specifically aimed at the daily assessment of patients who are involuntarily admitted.

*Special psychiatric intensive care unit patients are assessed on a daily basis. Patients are assessed on a daily basis. Then um, you sort of mark them off, in regards how mentally in terms of how they are like maybe aroused or whether they are generally settled on the ward, whether they are actually escalating.* (Group A)

Nurses mentioned however, that they only use these assessment tools as guidelines since they must be prepared for unexpected changes in the behaviour of their patients.

*So, although we use different types of risk assessment tools we don't particularly rely on them wholeheartedly. You know we've come to use as a guideline. Well of course we know this but at any point anything can happen, or it may not so you just have that in your thinking.* (Group D)

##### Communication among staff members

The nurses stated that effective communication among staff members around observed signs and symptoms of agitation in patients was crucial in the assessment of patients and the need for timely intervention and management.

*I think communication is key.* (Group D)

*We’re quite good at coming together as a team.* (Group A)

Communication could occur at any time during the shift and during handover. Debriefing sessions involving staff following an incident were also helpful in equipping nurses with better skills for recognising and managing agitation in the future. The outcomes of the ‘Targeted Risk Assessment’ were also said to be discussed within the team to initiate measures where necessary to minimise the risk of an escalation in agitated behaviour.

### Managing agitation

The management of agitation was organised into two themes: the type of interventions used and, the processes that support successful management.

#### Types of interventions

Nurses described the use of non-pharmacological and pharmacological interventions to manage agitation and how the two can be used in conjunction to manage agitation.

##### Non-pharmacological interventions

The use of de-escalation strategies were discussed in all focus groups as the first protocol used to manage agitation.

*… with us here, I think with any mental health services the first protocol we use is pretty much de-escalation. That’s the first line we go and you know, sit them, talk to them, depending on how they are.* (Group C)

*We go on de-escalation being the first line of call when we are trying to solve agitation on the ward.* (Group C)

In particular, nurses described ‘talking with patients’ as the first and least restrictive option to de-escalate the agitation and determine the cause.

*Well, non-pharmacological would be to talk to the patient to try and de-escalate, we do that all the time.* (Group B)

One nurse considered the staff were skilful in the use of verbal de-escalation since other more invasive measures such as seclusion were infrequently used.

*We are really quite talented in our de-escalation skills. Otherwise we would have a lot more seclusion than we actually do.* (Group C)

Nurses also described their use of other methods such as diversion and distraction to de-escalate agitation.

*We have things like weighted blankets, sometimes they’re useful. Like you mentioned distraction techniques, like maybe just suggesting they go and watch TV for a little bit or read a book …. Just maybe things they’d like to do like drawing or coloring in or write in journals, is often quite popular.* (Group A)

The use of restraint and/or seclusion was mentioned by three focus groups to manage agitation. One nurse described restraint as the last line of intervention in the secure ward, while other nurses described the need to use restraints under certain circumstances, including, when de-escalation strategies and the use of medication has not been effective or when patients refuse medication.

*We sometimes use restraint in patients who have been refusing medication. Then they might be refusing oral medication written by the doctor they need to have medication for treatment if they're under the act and that treatment can then be forced on them. Under the act and if they still refuse then we sometimes have to restrain somebody in a prone position to give them an injection.* (Group D)

##### Pharmacological interventions

While non-pharmacological interventions were described as the first approach, the respondents also reported there were occasions when medication was a necessity. For example, when de-escalation interventions have not been successful and the agitation was observed to be escalating, or when the agitation was related to substance withdrawal.

*They just escalate from 0-100, no matter how many times you try to talk to them. But then they start, you know, throwing chairs, doing whatever they do.* (Group C)

Nurses also described their use of the patient’s agitation and arousal chart to determine the level of agitation that necessitated a medication and the type of medication that could be administered.

#### Processes that support successful management

Nurses described a range of processes considered important to or underpinning how particular interventions were implemented and their success. Adaptability and flexibility on the part of nurses; adopting an individualised approach with patients; training and experience; rapport with patients, and patient involvement in care were discussed.

##### Adaptability and flexibility

When dealing with agitation, nurses described the need to adapt to the situation to ensure they remain calm and maintained an ‘adult’ perspective. Communicating in this manner was said to be key to effective de-escalation.

*Trying not to personalise somebody else's behavior to remain calm to stay in an adult mode, not become annoyed by the patient so that you’re not escalating the situation.* (Group D)

Two focus groups described the importance of being sufficiently flexible to consider alternative management strategies.

*So I guess it’s about identifying what could actually diffuse the patient agitation in the situation…..in that particular point in time….if that fails….then it could be about you know, trying to look at maybe things that have worked in the past if you know that particular patient.* (Group A)

One example described assisting a patient to fulfil their nicotine needs by providing a cigarette. This eased the episode of agitation and helped build rapport with the patient.

*With the immediate addiction needs like nicotine- one to one is not going to necessarily help with that. So, we really try hard to get some tobacco basically for them.* (Group D)

Nurses also described the need to be flexible and seek assistance from other staff to manage the patient’s agitation. For example, recognizing that another staff member might be more successful in de-escalating the patient’s agitation if they have established greater rapport. One younger nurse described the age gap between herself and older patients as sometimes impacting on her ability to deescalate a situation:

*…a lot of patients will go ‘ oh Ann (pseudonym), what do you know about life?’ and I will go and ask someone, you know, I have tried to talk to this patient, do you mind talking to that patient. So you find some times that they do listen better to someone else, you know. (Group C)*

##### Adopting an individualised approach

In relation to being adaptable, the nurses described the need to acknowledge the uniqueness and individual needs of patients and to tailor a management strategy that was best suited to them. There was an understanding that a ‘one size’ management response would not work for all patients.

*I find that I approach patients differently depending on their diagnosis, how long I know them, whether I have got any rapport and you know there's so many different forms of agitation as well. So, it just depends on so many factors, what works with one person may not work with a different person with a different type of agitation. So it's about knowing your patient as well as you can I guess to make those judgements*. (Group D)

The nurses were mindful that approaches that worked for one person may not also work for another.

##### Training and experience

Nurses described how training and experience influenced how they approached the management of an episode of agitation. They considered that being ‘experienced’ was a considerable advantage and a critical factor in the provision of care to manage agitated patients.

*Staff that have been around for a long time, know what to use and when to use it.* (Group C)

In relation to training, the nurses in all focus groups described mandatory completion of the *Professional Assault Response Training* (PART) program to deal with agitated or aggressive patients (which they are required to complete every 3 years). The training focused on the development of empathy and de-escalation techniques plus the use of physical restraint interventions. One focus group also mentioned undergoing Transactional Analysis training and receiving education and training sessions by the resident clinical psychologist. These education and training sessions helped with managing patients generally and in regard to agitation and assisted with their own personal development.

*Our clinical psychologist comes in and actually does different education sessions with us. And that’s invaluable. On deescalating as well and understanding why you are doing what you are doing.* (Group C)

Some nurses were critical however, of the training they had received and described how additional training and supervision to manage agitated patients was required.

*Regular clinical supervision, a formalized standard of clinical supervision, where on a particular day, you’ve got the chance to have one on one, but also like a group session on education, constantly educating staff on identifying triggers, how to manage situations…….best practice guidelines from around the world, and what works, what doesn’t and a standard formalized version of that would help*. (Group A)

##### Rapport

Building rapport with patients was acknowledged to be a key factor in de-escalating the behaviour of patients experiencing agitation. This could be achieved through honest and effective verbal communication with the patient, undertaking behaviours that eased the patient’s distress (such as the provision of a cigarette to deal with a patient’s nicotine addiction), and demonstrating a willingness to be adaptable. Nurses acknowledged that agitation could be more easily de-escalated if the patient trusted the nurse and associated ‘positive thoughts’ with the nurse.

*You can de-escalate to a certain extent, sit down and actually step through what is making them frustrated, rather than just going off getting medications straight away and build that rapport, build that trust because that in a nut shell is just so important. If you can, that is, instead of using medication every time. That should be the back-up, you know the rapport is actually meant to be the first, if you can do that.* (Group C)

*When they become agitated if you have built some rapport … it’s a useful tool to have later down the track they become agitated by other things they might remember the rapport.* (Group D)

##### Patient involvement

Nurses described the involvement of patients in the management of their agitation as important. The Unit’s ‘Coping and Safety Plan’ completed by the patient on admission was described as helpful for facilitating involvement, as well as a post-episode interview between the nurse and the patient to discuss the incident and the management of it.

*I asked if she could have anything that would help her calm down in regards to, in terms of medications and she didn’t want anything. After persuading her to eat, she agreed to a calmative medication in the form of a benzodiazepine…..maybe after thirty or so minutes, she had sort of like, calmed down.* (Group A)

## Discussion

### The recognition of agitation

Nurses described their experience of the recognition of agitation as a complex, dynamic process that drew on their professional skills and experience, assessment protocols operating within the mental health unit, and the contribution of patients. These resources and processes align with key elements of the person-centred nursing framework described by McCormack and McCance [17], namely, attributes of the nurse; attributes of the care environment, and attributes of the care process.

Nurses’ recognition of agitation was guided by their ‘experience’ and clinical understanding of the behavioural and verbal symptoms of agitation in patients (e.g., excessive restlessness, non-purposeful physical activity, pacing and shouting) which have been documented elsewhere [8, 23, 24]. In addition, nurses recognised the need to develop an awareness of the patient’s base level behaviour and unique signs of agitation as patients did vary in their experience and expression of agitation. These descriptions reflect aspects of professional competency which are among the many attributes McCormack and McCance [17] theorise nurses must possess to deliver person-centred care. Not only are nurses are expected to possess a requisite level of clinical knowledge and skill about a condition, but they must also recognise and respect the values and beliefs patients hold about their condition as an important source of information to be used in shared decision making [17].

Assessment tools (e.g., Brief Risk Assessment tool; Targeted Risk Assessment) were also used by nurses to assess agitation. This is consistent with recommended practice for clinicians to use standardised measures to objective assess patients for agitation [25]. While objective measures of agitation can be useful in the mental health setting, there is some difficulty determining which of the available scales is appropriate for use; scales can be either too general or too specific to a population to be useful [25]. Consistent with this limitation, the nurses described using the information from the agitation scales as a ‘guide’ only to be used in combination with their clinical observations and professional judgement.

Importantly, nurses acknowledged the diversity in the experience and expression of agitation among patients and sought to adopt an individualised approach to assessment. They reported using information provided by patients when they self-disclosed feeling agitated and other information about personal triggers for agitation reported in the Coping and Safety Plan completed by patients at admission. Through these practices nurses began to understand the patients’ beliefs, values and experiences about their condition and built rapport. This process is essential to person-centred nursing [17] as it aids the development of a therapeutic alliance between the nurse and patient for shared decision making about the patient’s health care [26]. The participating nurses in this study believed that engaging patients to share their experience of agitation effectively reduced the frequency and severity of agitation. This perception is consistent with previous research showing that patient engagement and the early recognition of an impending episode can prevent aggressive behaviours [27–30].

The description provided by the nurses of the recognition and assessment of agitation outlines an organisational framework and system within the mental health unit that enables them to draw upon and combine different sources of information about a patient to build an individualised understanding of their experience of agitation which can be used in shared decision making on how best to manage the condition. Care environments that promote diverse sources of knowledge and shared decision making optimise the opportunity to deliver person-centred nursing [17].

### The management of agitation

Nurses described how their management of agitation was based on combination of established principles and guidelines operating within the mental health unit, their training and experience, and input from patients. With certain exceptions, their management practices and processes align with reported best practice and the person-centred nursing framework and philosophies promoted by McCormack and McCance [17].

Nurses referred to a number of principles and guidelines to aid their management of agitation. The Coping and Safety Plan, completed by patients upon admission, provided them with information on potential triggers for agitation and strategies to manage patient agitation. Nurses also described how they encouraged patients to self-report becoming agitated so they could discuss management strategies. These practices reflect an individualised approach to managing patient agitation – as described by the nurses - and a willingness to engage patients to facilitate shared decision making over their care. Both of these processes are central to the delivery of effective person-centred care [17, 31].

The use of non-pharmacological strategies were identified by nurses as the first line of intervention to calm agitated patients, a position which has been widely endorsed in the literature as best-practice [4, 13, 32–34]. This typically involved talking with the patient to determine the causes of their agitation and how it could be best managed. This approach was judged by nurses to be effective in de-escalating agitation and building rapport. From a person-centred care perspective, de-escalation strategies provide the nurse with an opportunity to demonstrate empathy and respect toward the patient and build on the therapeutic relationship that is critical to person-centred care [17].

Nurses also described the use of seclusion and restraint as a ‘last line’ strategy to manage patients when less restrictive, non-coercive interventions had not been successful or were inappropriate. Restraint was said to be used when de-escalation strategies and even medication had not been effective or when patients refused prescribed medication which they were required to take because they were an involuntary patient. The use of restraint in these circumstances is consistent with the belief that it can guarantee patient safety and required care, particularly when the patient is agitated or suicidal [35]. However, the use of restraint is at odds with the global movement for a cessation of its use [36, 37]. The use of restraint can result in physical injuries [4, 15] and emotional distress for staff and patients [14]. Its use may also impact on the effective delivery of person-centred care as it is a coercive strategy [35] that has the potential to negatively impact the important therapeutic relationship between staff and patients.

## Limitations

There are a number of issues which potentially limit the representativeness and transferability of the findings. Firstly, the study was conducted in the mental health unit of one hospital. Secondly, the focus groups were conducted on 1 day only with staff rostered on that day and therefore excludes other non-rostered staff who work in the unit. Due to staffing limitations, the focus group sessions were also limited to a maximum of 30 min which may have limited the depth and detail of the responses provided by participants. Finally, there was no opportunity for participants to provide feedback on and validate the transcribed accounts of their focus group session.

## Conclusions

This study explored how mental health nurses recognise and manage agitation and how these processes align with best practice and person-centred care principles. Nurses described combining their clinical knowledge, assessment protocols and training with information from patients to make an individualised assessment of agitation. Nurses adopted an individualised approach to management by engaging patients in decisions about their care. In keeping with best practice recommendations, de-escalation strategies were the first choice option for management, though nurses also described using both coercive and medication under certain circumstances. When the findings are examined from the perspective of patient-centred care, there is good reason to conclude that the mental health unit’s organisation and the care provided by its staff aligns with various elements of the patient-centred nursing framework proposed by McCormack and McCance [17] and person-centred care more generally.

## Data Availability

The focus group transcript data are available from the corresponding author on request.

## Abbreviations

PPC: Person-centred care
TA: Thematic analysis
